# Follicle-stimulating hormone responsiveness in antral follicles from aryl hydrocarbon receptor knockout mice

**DOI:** 10.1186/1477-7827-11-26

**Published:** 2013-04-01

**Authors:** Isabel Hernández-Ochoa, Liying Gao, Jackye Peretz, Mallikarjuna S Basavarajappa, Stacey L Bunting, Bethany N Karman, Tessie Paulose, Jodi A Flaws

**Affiliations:** 1 Department of Comparative Biosciences, University of Illinois, Urbana-Champaign, Illinois 61802, USA; 2 Departamento de Toxicología, Cinvestav-IPN, México, D F 07360, México

**Keywords:** Aryl hydrocarbon receptor, Ovary, Follicle growth, FSH, Inhibin A, Steroidogenesis

## Abstract

**Background:**

Previous studies have demonstrated that pre-pubertal aryl hydrocarbon receptor knockout (AHRKO) mice have slow antral follicle growth and reduced capacity to produce estradiol compared to wild-type (WT) mice. Although previous studies have suggested that this is likely due to a reduced ability of the AHRKO follicles to respond to follicle-stimulating hormone (FSH), this possibility was not directly tested. Thus, the goal of these studies was to test the hypothesis that low FSH responsiveness is responsible for the slow growth and reduced estradiol production observed in pre-pubertal AHRKO *versus* WT antral follicles.

**Methods:**

Antral follicles from WT and AHRKO mice were cultured with varying amounts of FSH (0–15 IU/mL) for up to 7 days, and subjected to measurements of growth, FSH receptor and steroidogenic regulator expression, sex steroid hormone levels, and inhibin beta-A expression. General linear models (GLM) for repeated measures were used to compare follicle diameters over time among treatments. If the global tests from GLM were significant, Tukey’s tests were used for pairwise comparisons. Remaining comparisons among groups were performed using one-way analysis of variance followed by Tukey’s *post hoc* test.

**Results:**

The results indicate that FSH stimulated growth in both WT and AHRKO follicles, but that high levels of FSH (10–15 IU/mL) were required for AHRKO follicles to reach maximal growth, whereas lower levels of FSH (5 IU/mL) were required for WT follicles to reach maximal growth. Further, FSH stimulated expression of FSH receptor, steroidogenic factors, and inhibin beta-A as well as production of steroid hormones in both WT and AHRKO follicles, but the degree of stimulation differed betw een WT and AHRKO follicles. Interestingly, FSH treatment increased expression of FSH receptor, some steroidogenic regulators, inhibin beta-A, and steroid hormone production more in AHRKO follicles compared to WT follicles.

**Conclusions:**

Collectively, these data suggest that the slow growth, but not reduced steroidogenesis in AHRKO follicles, is due to their reduced ability to respond to FSH compared to WT follicles. These data also suggest that the AHR may contribute to the ability of FSH to stimulate proper follicle growth, but it may not contribute to FSH-induced steroidogenesis.

## Background

The aryl hydrocarbon receptor (AHR) is a ligand-activated transcription factor [1] that has been shown to have important biological roles in ovarian function [2-8]. Specifically, previous studies indicate that pre-pubertal aryl hydrocarbon receptor knockout (AHRKO) mice have slower antral follicle growth and a reduced capacity to produce estradiol compared to wild-type (WT) mice [2-8]. Further, the slow growth and reduced estradiol production is only observed in pre-pubertal AHRKO mice, but not in sexually mature AHRKO mice [8].

One possible explanation for the slow follicle growth and reduced production of estradiol in pre-pubertal AHRKO mice compared to pre-pubertal WT mice may be that pre-pubertal AHRKO mice have a reduced capacity to respond to follicle-stimulating hormone (FSH) compared to pre-pubertal WT mice. FSH is critically important for promoting estradiol production in granulosa cells, and estradiol is the hormone that directly stimulates growth in antral follicles [9,10]. Given that the levels of FSH are generally low prior to puberty and dramatically rise after puberty, it is possible that AHRKO mice are able to overcome their potentially low ability to respond to FSH prior to puberty by the presence of higher FSH levels after they become sexually mature. These possibilities are supported by previous studies that show that AHRKO follicles have reduced mRNA expression of FSH receptors (*Fshr*) as well as a reduced number of FSH binding sites compared to WT follicles [5,8]. Further, they are supported by studies indicating that the AHR and FSH pathways may interact to regulate transcription of the *Fshr* because the AHR binds to aryl hydrocarbon response elements in the promoter regions of the *Fshr* and an E-box binding site in the mouse ovary [5,11].

If pre-pubertal AHRKO follicles are less responsive than pre-pubertal WT follicles to FSH, this may lead to reduced estradiol biosynthesis and slow follicle growth. This is because binding of FSH to FSHR promotes cytochrome P450, family 19, subfamily A, polypeptide 1 (*CYP19A1*) expression [12], the enzyme that converts theca cell-produced androgens (*i.e*., dehydroepiandrosterone and androstenedione) into estrogens (*i.e*., estradiol and estrone) [9,10,13], and the estrogens then stimulate proliferation of granulosa cells to promote follicle growth [9,14]. Thus, the current study was designed to test the hypothesis that low FSH responsiveness is responsible for the slow growth and reduced estradiol production observed in pre-pubertal AHRKO *versus* pre-pubertal WT antral follicles.

To test this hypothesis, antral follicle growth was compared in follicles from pre-pubertal WT and AHRKO mice in response to varying concentrations of FSH. As balanced steroid hormone synthesis is crucial for antral follicle growth [9], the levels of steroid hormones and the expression of factors that regulate steroidogenesis were also compared in WT and AHRKO follicles cultured with FSH. Further, studies also show that FSH regulates the levels of inhibin beta-A (INHBA) in antral follicles during granulosa cell differentiation [15-17]; and that reduced levels of *Ahr* mRNA are related to high levels of *Inhba* mRNA in antral follicles [18]. Thus, the levels of *Inhba* mRNA were compared in WT and AHRKO follicles cultured with varying concentrations of FSH.

## Methods

### Chemicals

Fetal bovine serum (FBS) was obtained from Atlanta Biologicals (Lawrenceville, GA). Human recombinant FSH was obtained from Dr. A.F. Parlow from the National Hormone and Peptide Program (Harbor-UCLA Medical Center, Torrance, CA). Penicillin, streptomycin, ITS (insulin, transferrin, selenium), antibiotic antimycotic solution, and ampicillin were obtained from Sigma-Aldrich (St. Louis, MO). Alpha-minimal essential medium (α-MEM) was obtained from Invitrogen (Carlsbad, CA).

### Animals

The AHRKO mice were generated by Schmidt *et al.*[19] and are in C57BL/6J background, along with their WT littermates. Only homozygous mice (WT and AHRKO) were used from breeding colonies maintained by our laboratory at the University of Illinois at Urbana-Champaign, Veterinary Medicine Animal Facility. Mice were provided with food and water for *ad libitum* consumption, and maintained in a temperature and light controlled room (24 ± 1°C, 12 h daylight/12 h dark cycle) with 35 ± 4% relative humidity. Genetic screening was performed using ear tissue punches as previously described [5,6]. Female WT and AHRKO mice were euthanized by carbon dioxide inhalation. The ovaries were removed and early antral follicles were isolated as described below. All animal care, euthanasia, and tissue collection were approved by the Institutional Animal Use and Care Committee at the University of Illinois.

### Antral follicle isolation

Ovaries were removed from pre-pubertal WT and AHRKO mice on post-natal day (PD) 30–32 (3–6 mice per genotype per experiment), placed in α-MEM, and cleaned of both interstitial tissue and small follicles using fine watch maker forceps under a dissecting microscope. WT and AHRKO mice on PDs 30–32 were considered to be of a pre-pubertal age because of their lack of a vaginal opening and lack of regular estrous cyclicity. Approximately 20–30 early antral follicles (260–400 μm) were mechanically isolated per ovary, pooled, and randomly assigned by genotype to follicle culture for *in vitro* follicle growth evaluation as described below.

### Measurement of follicle growth

Follicle growth was evaluated in cultured antral follicles as previously described [6,20]. Briefly, early antral follicles from WT and AHRKO mice on PDs 30–32 (3–6 mice per genotype per experiment) were placed individually in 96-well culture plates with 150 μL α-MEM, which was immediately replaced with 150 μL of supplemented α-MEM containing 5% FBS, 1% ITS (10 ng/mL insulin, 5.5 ng/mL transferrin, and 5.5 ng/mL selenium), 100 U/mL penicillin, 100 mg/mL streptomycin, and 0, 5, 10 or 15 IU/mL FSH. Follicles were then incubated for 7 days at 37°C in 95% air, 5% carbon dioxide, and humidity saturated. Follicle growth patterns were examined by measuring follicle diameters in perpendicular axes every 24 h using an inverted microscope equipped with a calibrator ocular micrometer as described by Miller *et al.*[20]. Culture media were collected on days 3 and 7 and subjected to measurements of steroid hormone levels as described below. Follicles were discarded from cultures if they were dark in appearance or if they could not retain their oocyte enclosed within the granulosa cell mass. At least three separate cultures per FSH treatment group in WT and AHRKO mice were performed. Each experiment contained 10–16 follicles per treatment. The sizes of follicles per treatment were averaged in each experiment and then data across separate cultures were averaged. At the end of culture, follicles were snap-frozen and stored at −80°C until quantitative PCR (qPCR) analyses as described below.

### Measurement of steroid hormone levels

Culture media samples were subjected to enzyme linked immunoassays (ELISA) using kits for measurement of progesterone and androstenedione (DRG International Inc., Mountainside, NJ), dehydroepiandrosterone (DHEA; Alpco Diagnostics, Salem, NH), and estradiol (Calbiotech, Spring Valley, CA). All samples from 5–15 IU/mL FSH treatments were diluted 1:2 (v/v) for androstenedione and 1:5 (v/v) for progesterone assays. Samples collected on days 3 and 7 were diluted 1:10 (v/v) and 1:100 (v/v), respectively, for estradiol assays. Hormone levels were measured on day 3 because this is a time at which media need to be changed in the culture system to maximize follicle growth. Hormone levels were measured on day 7 because this was the last day of culture. Then, a sum of day 3 and day 7 hormone levels was made to represent total hormone production by follicles as they grew in culture. All ELISA procedures were performed according to the manufacturer’s protocol. α-MEM medium was used as background control. Lyphochek Immunoassay Plus controls (Bio-Rad laboratories, Inc.) containing known amounts of specific hormones were included as positive controls in every assay. All samples were run in duplicate, and values were calculated by multiplying by the corresponding dilution factor. The analytical sensitivities, as determined by the ELISA kit manufacturers, were 0.045 ng/mL for progesterone, 0.005 ng/mL for DHEA, 0.019 ng/mL for androstenedione, and 10 pg/mL for estradiol. No samples were below the limit of detection. Intra-assay and inter-assay coefficients of variation for all assays were <10%.

### Quantitative real-time polymerase chain reaction (qPCR)

Total RNA (1–2 μg) from pooled antral follicles was extracted using the RNeasy Mini Kit (Qiagen, Inc., Valencia, CA) and then converted to cDNA using iScript cDNA synthesis kit (Bio-Rad Laboratories Inc., Hercules, CA) according to the manufacturers’ protocols. The cDNA was amplified by qPCR as previously described [21] using a CFX96 Real-time System C1000 Thermal Cycler (Bio-Rad Laboratories Inc., Hercules, CA) and accompanying software according to the manufacturer’s instructions. To allow analysis of the amount of cDNA in the exponential phase, a standard curve from five serial dilutions was generated using cDNA from a pool of WT and AHRKO antral follicles. Specific qPCR primers (Integrated DNA Technologies, Inc, Coralville, IA) for the genes of interest and annealing temperatures are listed in Table 1. SsoFast EvaGreen Supermix (Bio-Rad Laboratories Inc., Hercules, CA) was used as dye for all qPCR analyses. A melting curve was generated at 55–90°C to confirm the generation of a single product, and PCR products were loaded in 3% agarose gel to confirm the product size according to Table 1. Beta actin (*Actb*) was used for each sample as an internal control. Relative transcript amount was calculated by a mathematical model developed by Pfaffl [22]. Briefly, the method calculates the relative expression ratio of the target gene based on the amplification efficiency of each amplicon and the ΔCt of the treated samples *versus* the vehicle control. These ratios were then compared to the expression of the reference gene *Actb*.

**Table 1:** Primers used in real-time qPCR analysis

**Gene name**	**Gene symbol**	**Primer sequence (5’-3’)**	**Annealing temperature (°C)**	**Band (bp)**	**GenBank accession no.**
Beta actin	*Actb*	F: ctggcaccacaccttctac	55.0	238	NM_007393
		R: gggcacagtgtgggtgac			
Cytochrome P450, family 11, subfamily A, polypeptide 1	*Cyp11a1*	F: agatcccttcccctggcgacaatg	60.0	192	NM_019779
		R: cgcatgagaagagtatcgacgcatc			
Cytochrome P450, family 17, subfamily A, polypeptide 1	*Cyp17a1*	F: ccaggacccaagtgtgttct	56.0	250	NM_007809
		R: cctgatacgaagcacttctcg			
Cytochrome P450, family 19, subfamily A, polypeptide 1	*Cyp19a1*	F: catggtcccggaaactgtga	56.0	187	NM_007810
		R: gtagtagttgcaggcacttc			
Follicle stimulating hormone receptor	*Fshr*	F: gcagatgtgttctccaacctacc	61.0	172	NM_013523
		R: ggagagactggatcttgtgaaagg			
Hydroxy-delta-5-steroid dehydrogenase, 3 beta- and steroid delta-isomerase 1	*Hsd3b1*	F: caggagaaagaactgcaggaggtc	59.5	280	NM_008293
		R: gcacacttgcttgaacacaggc			
Steroidogenic acute regulatory protein	*Star*	F: cagggagaggtggctatgca	57.0	262	NM_011485
		R: ccgtgtcttttccaatcctctg			
Inhibin, beta-a	*Inhba*	F: tcacctttgccgagtcaggc	59.0	97	NM_008380
		R: ccacacttctgcacgctcca			

F, forward; R, reverse.

### Statistical analysis

The data were analyzed using SAS 9.2 (Statistical Analysis System Institute, Inc.). General linear models (GLM) for repeated measures were used for comparisons over time in follicle cultures. If the global tests from GLM were significant, Tukey’s tests were used for pairwise comparisons. Multiple comparisons between experimental groups were conducted on data obtained from at least three independent experiments using one-way analysis of variance (ANOVA) followed by Tukey’s *post hoc* test. Data were expressed as means ± SEM. Statistical significance was assigned at p ≤ 0.05.

## Results

### Effects of FSH on *in vitro* antral follicle growth

**Figure 1:**
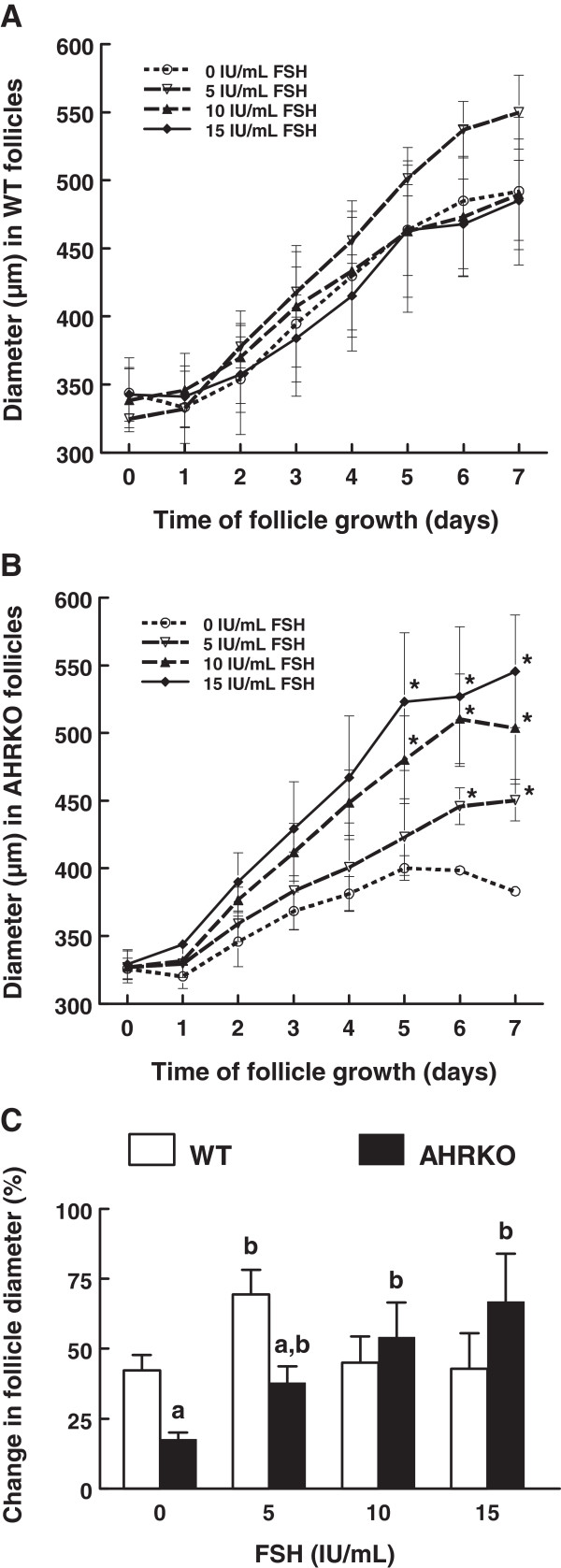
**Comparison of follicle growth in response to FSH treatment in WT and AHRKO follicles.** Early antral follicles from pre-pubertal WT (**A**) and AHRKO (**B**) ovaries were cultured in supplemented media in the presence of 0–15 IU/mL FSH for 7 days. Growth of follicles was monitored every 24 h by measuring follicle diameter in perpendicular axes and plotted as follicle diameters. (**C**) Total percent change in WT and AHRKO follicle diameters from day 0 to day 7 of culture. Graphs represent the mean ± SEM from three separate cultures, each with 10–16 follicles per genotype. The asterisk (*) above the dotted line indicates significant differences (p ≤ 0.05) from 0 IU/mL FSH at the same day of follicle growth, using a GLM followed by Tukey’s test. The letter “a” above the bars indicates significant difference (p ≤ 0.05) from WT follicles within the same FSH treatment group and the letter “b” above the bar indicates significant difference (p ≤ 0.05) from 0 IU/mL FSH in the same genotype, using one-way ANOVA followed by Tukey’s test as a *post hoc* test.

To evaluate whether WT and AHRKO follicles respond similarly to FSH in terms of follicle growth, early antral follicles from WT and AHRKO mice were cultured with vehicle (supplemented α-MEM; 0 IU/mL FSH) or increasing concentrations of FSH (5–15 IU/mL; Figure 1) and their diameter measured every 24 h for a total culture time of 7 days. Wild-type antral follicles treated with 0, 5, 10 or 15 IU/mL FSH were able to grow to a similar degree over the 7-day culture period (Figure 1A). AHRKO antral follicles significantly grew starting at day 5 in the presence of 10 or 15 IU/mL FSH or they grew starting at day 6 in the presence of 5–15 IU/mL FSH, as compared to AHRKO follicles with no FSH treatment (Figure 1B). When change in follicle diameter from day 0 to day 7 of culture was compared in WT and AHRKO follicles (Figure 1C), WT follicles with no FSH treatment grew more than AHRKO follicles with no FSH treatment and WT follicles treated with 5 IU/mL FSH grew more than AHRKO follicles treated with 5 IU/mL FSH treatment. However, WT and AHRKO follicles grew to a similar degree in response to higher concentrations of FSH (10 and 15 IU/mL).

### Effects of FSH on theca cell derived steroidogenic factors and steroid hormones

To evaluate whether WT and AHRKO follicles respond similarly to FSH in terms of estradiol production, levels of steroidogenic enzymes and sex steroid hormones were compared in WT and AHRKO follicles in response to FSH. Normal estradiol production requires expression of steroidogenic factors and production of sex steroid hormones in both the thecal cells and granulosa cells of antral follicles [23-25]. Thus, we first compared thecal cell-derived expression of steroidogenic factors and sex steroid hormone levels in WT and AHRKO follicles in response to FSH. Specifically, we examined mRNA levels of steroidogenic acute regulatory protein (*Star*), cytochrome P450, family 11, subfamily A, polypeptide 1 (*Cyp11a1*), cytochrome P450, family 17, subfamily a, polypeptide 1 (*Cyp17a1*) and hydroxy-delta-5-steroid dehydrogenase, 3 beta- and steroid delta-isomerase 1 (*Hsd3b1*) because these factors are in thecal cells and they are transcriptionally regulated [23,24].

**Figure 2:**
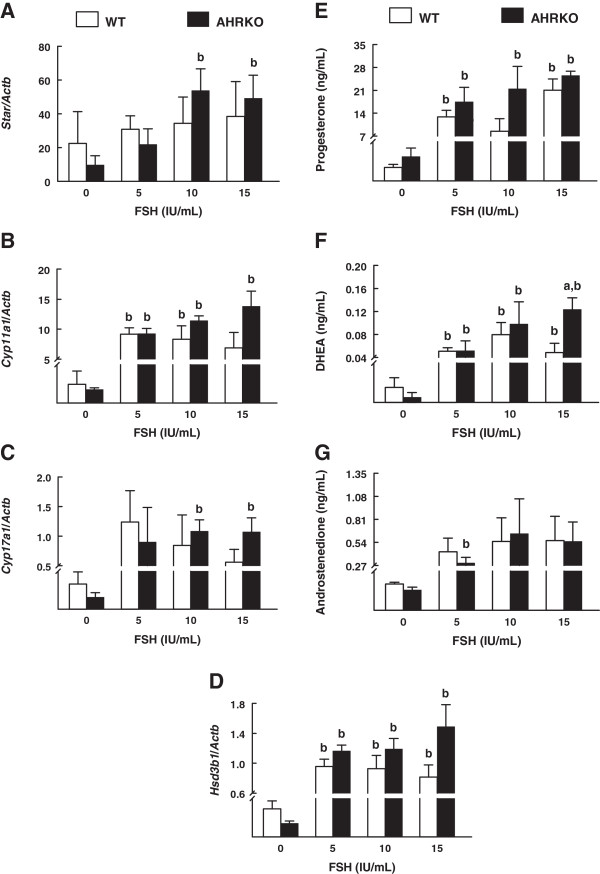
**Comparison of theca-cell derived factors in response to FSH treatment in WT and AHRKO follicles.** Early antral follicles from pre-pubertal WT and AHRKO ovaries were isolated and cultured in supplemented media in the presence of 0–15 IU/mL FSH for 7 days. At the end of culture, follicles were pooled per treatment group and genotype, subjected to extraction of RNA and then processed for qPCR analysis of *Star* (**A**), *Cyp11a1* (**B**), *Cyp17a1* (**C**)*,* or *Hsd3b1* (**D**). Each gene was expressed as a mean relative expression ratio normalized to *Actb* as a loading control. Media samples from individual follicles were obtained from cultures and subjected to ELISAs to measure levels of progesterone (**E**), DHEA (**F**) and androstenedione (**G**). Each bar represents the mean ± SEM from three separate cultures per genotype at each selected treatment and from 10–18 follicles per treatment. The letter “a” above the bars indicates significant difference (p ≤ 0.05) from WT follicles within the same FSH treatment and the letter “b” above the bars indicates significant difference (p ≤ 0.05) from 0 IU/mL FSH in the same genotype, using one-way ANOVA followed by Tukey’s test as a *post hoc* test.

In both WT and AHRKO follicles, mRNA levels for theca-cell derived factors increased with either low or high concentrations of FSH (Figures 2A-2D). Further, both WT and AHRKO follicles had similar levels of *Star* (Figure 2A), *Cyp11a1* (Figure 2B), *Cyp17a1* (Figure 2C), and *Hsd3b1* mRNA regardless of the FSH concentration (Figure 2D).

Normal estradiol production also requires the production of progesterone, DHEA and androstendione by thecal cells in the follicle. Thus, we evaluated whether WT and AHRKO follicles respond similarly to FSH in terms of production of progesterone, DHEA, and androstendione. Theca cell-derived progesterone (Figure 2E) and DHEA (Figure 2F) significantly increased in media samples from WT and AHRKO follicles treated with each concentration of FSH (5–15 IU/mL) compared to media samples from follicles of the same genotype with no FSH treatment. Theca cell-derived androstenedione (Figure 2G) significantly increased only in media samples from AHRKO follicles treated with 5 IU/mL FSH compared to media samples from AHRKO follicles with no FSH treatment.

### Effects of FSH on steroidogenic factors and steroid hormones in granulosa cells

**Figure 3:**
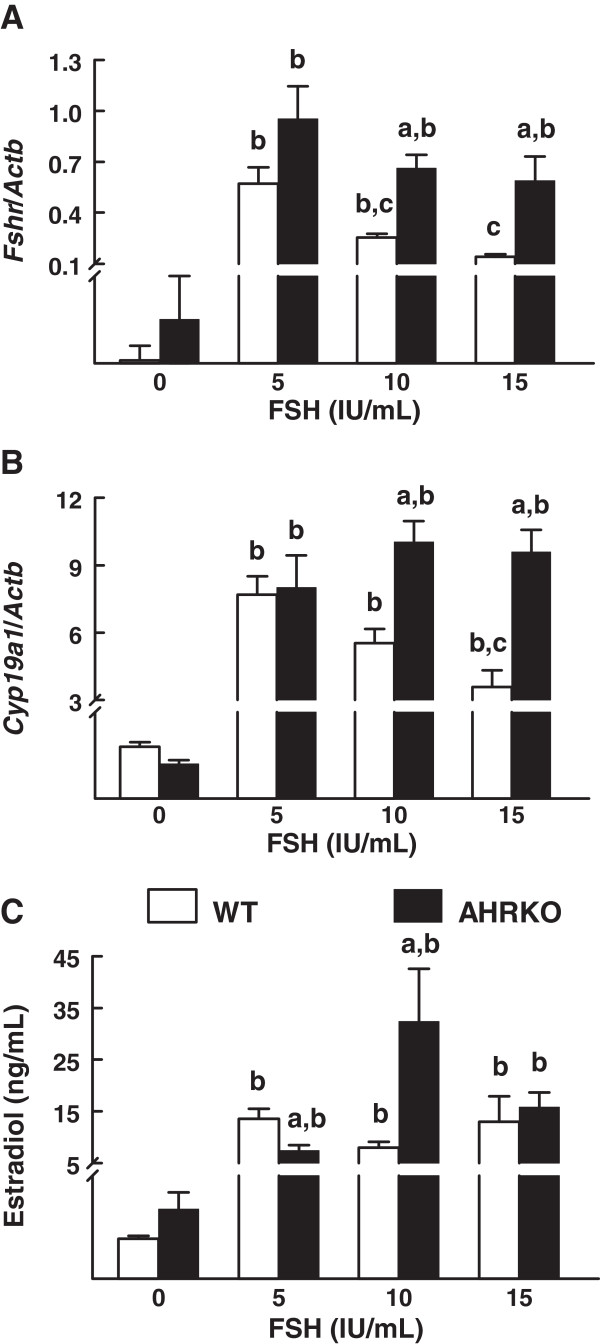
**Comparison of granulosa-cell derived factors in response to FSH treatment in WT and AHRKO follicles.** Early antral follicles from pre-pubertal WT and AHRKO ovaries were isolated and cultured in supplemented media in the presence of 0–15 IU/mL FSH for 7 days. At the end of culture, follicles were pooled per treatment group and genotype, subjected to extraction of RNA and then processed for qPCR analysis of *Fshr* (**A**) and *Cyp19a1* (**B**). Each gene was expressed as a mean relative expression ratio normalized to *Actb* as a loading control. Media samples from individual follicles were obtained from cultures and subjected to ELISAs to measure levels of estradiol (**C**). Each bar represents the mean ± SEM from three separate cultures per genotype at each selected treatment and from 10–18 follicles per treatment. The letter “a” above the bars indicates significant difference (p ≤ 0.05) from WT follicles within the same FSH treatment, the letter “b” above the bars indicates significant difference (p ≤ 0.05) from 0 IU/mL FSH in the same genotype and letter “c” above the bar indicates significant difference (p ≤ 0.05) from WT follicles treated with 5 IU/mL FSH, using one-way ANOVA followed by Tukey’s test as a *post hoc* test.

Normal estradiol production requires granulosa cell expression of *Fshr* and conversion of thecal-derived androgens to estradiol in the granulosa cells. Further, FSH may regulate the expression of its own receptor (*Fshr*) in granulosa cells [9,26-29] and binding of FSH to FSHR leads to increased transcription of *Cyp19a1* in granulosa cells [12], the enzyme that converts theca cell-derived androgens into estradiol. Thus, we evaluated whether FSH responsiveness is reduced in AHRKO *versus* WT follicles by altering granulosa cell-derived factors. Specifically, at the end of the 7-day culture period with increasing concentrations of FSH, follicles were processed for analysis of gene expression of *Fshr* and *Cyp19a1* by qPCR. Treatment with FSH increased expression of *Fshr* in both WT and AHRKO follicles, but FSH (10 and 15 IU/mL) significantly increased expression of the *Fshr* to a greater degree in AHRKO follicles compared to WT follicles (Figure 3A). Further, down-regulation of *Fshr* expression occurred in WT, but not AHRKO follicles (Figure 3A). Levels of *Cyp19a1* mRNA were similar in WT and AHRKO follicles treated with 5 IU/mL FSH, but higher in AHRKO follicles treated with 10 or 15 IU/mL FSH compared WT follicles (Figure 3B). Further, levels of *Cyp19a1* mRNA were higher in WT and AHRKO follicles treated with 5, 10, or 15 IU/mL FSH compared to follicles of the same genotype with no FSH treatment. In contrast, WT follicles treated with 15 IU/mL FSH had lower levels of *Cyp19a1* mRNA than those in WT follicles treated with 5 IU/mL FSH (Figure 3B).

Next, because estradiol is regulated by FSH, produced by granulosa cells, and promotes follicle growth [9,10,13,14], levels of estradiol were measured in media samples collected from WT and AHRKO follicles treated with increasing concentrations of FSH. Levels of estradiol were similar in media from WT and AHRKO follicles cultured in the presence of 15 IU/mL FSH (Figure 3C). However, levels of estradiol were decreased in media from AHRKO follicles in the presence of 5 IU/mL FSH and increased in media from AHRKO follicles cultured in the presence of 10 IU/mL FSH compared to media from WT follicles (Figure 3C). Further, significant increases in levels of estradiol were observed in WT and AHRKO follicles cultured in the presence of 5, 10, or 15 IU/mL FSH compared to follicles cultured in the presence of 0 IU/mL FSH in the same genotype (Figure 3C).

### Effects of FSH on expression of *Inhba*

**Figure 4:**
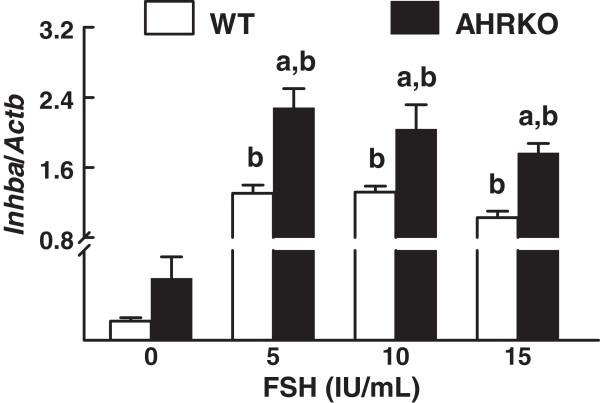
**Comparison of *Inhba *mRNA levels in response to FSH treatment in WT and AHRKO follicles.** Early antral follicles from pre-pubertal WT and AHRKO ovaries were isolated and cultured in supplemented media in the presence of 0–15 IU/mL FSH for 7 days. At the end of culture, follicles were pooled per treatment group and genotype, subjected to extraction of RNA and then processed for qPCR analysis of *Inhba*, which is expressed as a mean relative expression ratio normalized to *Actb* as a loading control. Each bar represents the mean ± SEM (n=3-6 mice per genotype and 10–16 follicles at each selected treatment). The letter “a” above the bars indicates significant difference (p ≤ 0.05) from WT follicles within the same FSH treatment, and the letter “b” above the bar indicates significant difference (p ≤ 0.05) from 0 IU/mL FSH in the same genotype, using one-way ANOVA followed by Tukey’s test as a *post hoc* test.

To further evaluate FSH responsiveness in AHRKO follicles *versus* WT follicles, levels of *Inhba* mRNA were compared in WT and AHRKO follicles at the end of culture (Figure 4). We elected to focus on *Inhba* expression as a marker of FSH responsiveness because this follicular factor is directly regulated by FSH through binding to FSHR [15,16,25,26]. Increasing concentrations of FSH (5–15 IU/mL) resulted in significantly increased levels of *Inhba* mRNA to a greater degree in AHRKO follicles than in WT follicles (Figure 4).

## Discussion

While previous studies indicate that pre-pubertal AHRKO antral follicles have slow growth and reduced estradiol production compared to WT follicles [2-6], it was not known whether these alterations were due to low FSH responsiveness in AHRKO *versus* WT follicles. To investigate this issue, we used an isolated follicle culture system to compare the direct effects of FSH on pre-pubertal WT and AHRKO antral follicles. Our main findings suggest that AHRKO follicles are less responsive to FSH-induced follicle growth than WT follicles because higher levels of FSH are required for AHRKO follicles to reach the same degree of growth as WT follicles. Surprisingly, the response of WT and AHRKO follicles to FSH in terms of steroid hormone production and steroidogenic regulator expression did not follow the response of follicle growth to FSH. Instead, FSH stimulated production of sex steroid hormones and steroidogenic regulators to a similar or greater degree in AHRKO follicles compared to WT follicles. These data suggest that the AHR may contribute to FSH-stimulated follicle growth, but it may not contribute to the ability of FSH to stimulate steroidogenesis.

Based on lower mRNA expression of *Fshr* as well as reduced binding sites for FSH in AHRKO ovaries compared to WT ovaries already shown in previous studies [5,8] and the higher FSH levels needed in our experiments for AHRKO follicles to reach WT growth levels, we initially expected that FSH would not be able to stimulate expression of *Fshr* to the same degree in AHRKO follicles as it does in WT follicles. However, our data indicate an opposite scenario in which FSH increases expression of *Fshr* to a greater level in AHRKO follicles compared to WT follicles. Further, our data indicate that the down-regulation of *Fshr* expression upon rising levels of FSH occurred in WT follicles, but not in AHRKO follicles. Because previous studies indicate that FSH can increase its own receptor, leading to increased ability of follicles to respond to FSH [9,27-30], our data suggest that the reduced levels of *Fshr*, and thus, the reduced capacity of AHRKO follicles to respond to FSH are not due to an inability of FSH to stimulate expression of its own receptor.

It is unclear how rising levels of FSH down-regulate expression of the *Fshr* in WT follicles, but not in AHRKO follicles. Previous studies indicate that low levels of FSH may favor increased *Fshr* expression in granulosa cells, whereas high levels of FSH suppress *Fshr* expression in granulosa cells [29]. It is possible that differences in *Fshr* transcript levels in WT and AHRKO follicles treated with rising levels of FSH may be explained by findings suggesting that the AHR is recruited to the *Fshr* promoter to transcribe the *Fshr* in WT, but not AHRKO follicles [5]. As FSH levels increase in WT follicles, the high FSH levels may lead to down-regulation of the *Fshr*. In AHRKO follicles, however, there is no active AHR; therefore, *Fshr* transcription may occur independently of the AHR. The AHR has been suggested to be indispensable for proper transcription of *Cyp19a1* in the ovary [4]. In our experiments, however, *Cyp19a1* as well as *Inhba* transcription followed a very similar pattern in response to FSH in AHRKO follicles compared to WT follicles, suggesting that FSH regulation of *Cyp19a1* and *Inhba* may not directly require the presence of the AHR. Instead, they may require proper transcription of *Fshr*.

Our experiments used a minimal concentration of 5 IU/mL FSH, which has been shown to be an essential dose for sustaining *in vitro* follicle growth [6,20]. Because the physiological doses of FSH that are required to promote growth in antral follicles are unknown, our use of a range of doses of FSH allowed us to compare FSH responsiveness in WT and AHRKO follicles. Unexpectedly, follicles were still able to grow to some degree when FSH was omitted from the culture. This finding is supported by previous studies that have demonstrated that FSHR knockout mice can develop pre-antral follicles up to the early antral follicle stage, but not further [31-33]. In addition, follicles cultured with no FSH treatment during the antral phase have limited granulosa cell differentiation in terms of their ability to produce estradiol [34]. As shown in our experiments, estradiol production and hormone precursors in follicles cultured with no FSH treatment were lower than those cultured with FSH treatment regardless of genotype. Since our *in vitro* culture uses a luteinizing hormone (LH)-free culture medium, androgen levels may be the result of constitutive androgen production by the theca cells, which provided sufficient substrate for estradiol production [34].

## Conclusions

In summary, our data indicate that higher levels of FSH are required to stimulate follicle growth in AHRKO follicles compared to WT follicles. In contrast, FSH is able to stimulate expression of steroidogenic factors*, Fshr*, and *Inhba* as well as production of some sex steroid hormones to a similar or greater degree in AHRKO follicles compared to WT follicles. Collectively, these data suggest that the AHR may contribute to the ability of FSH to stimulate proper follicle growth, but it may not contribute FSH-stimulated steroidogenesis.

## Abbreviations

ACTB: Actin beta; AHR: Aryl hydrocarbon receptor; AHRKO: Aryl hydrocarbon receptor knockout; α-MEM: Alpha-minimal essential media; ANOVA: Analysis of variance; CYP11A1: Cytochrome P450, family 11, subfamily A, polypeptide 1; CYP17A1: Cytochrome P450, family 17, subfamily A, polypeptide 1; CYP19A1: Cytochrome P450, family 19, subfamily A, polypeptide 1; DHEA: Dehydroepiandrosterone; ELISA: Enzyme-linked immunosorbant assay; FBS: Fetal bovine serum; FSH: Follicle-stimulating hormone; FSHR: Follicle-stimulating hormone receptor; GLM: General linear model; HSD3B1: Hydroxy-delta-5-steroid dehydrogenase, 3 beta- and steroid delta-isomerase 1; INHBA: inhibin beta-a; ITS: Insulin, transferrin, selenium; LH: Luteinizing hormone; PD: Post-natal day; qPCR: Quantitative polymerase chain reaction; STAR: Steroidogenic acute regulatory protein; WT: Wild-type.

## Competing interests

The authors declare that they have no competing interests.

## Authors' contributions

IHO designed the experiments, conducted the experiments, and wrote the initial draft of the manuscript. LG, JP, MB, SB, BK, and TP helped conduct the follicle isolations, cultures, and qPCR. BK also helped make the figures. JF helped design the experiments, edited drafts of the manuscript, and helped with data analysis and interpretation. She also obtained funding for the project. All authors read and approved the final manuscript.
